# Effects of Atorvastatin on Resting and Peak Exercise Blood Pressure among Normotensive Men and Women

**DOI:** 10.1155/2014/720507

**Published:** 2014-11-18

**Authors:** Amanda L. Zaleski, Marianne L. Mentch, Linda S. Pescatello, Beth A. Taylor, Jeffrey A. Capizzi, Adam S. Grimaldi, Priscilla M. Clarkson, Stephanie A. Moeckel-Cole, Stuart R. Chipkin, Justin Keadle, Charles Michael White, Paul D. Thompson

**Affiliations:** ^1^Henry Low Heart Center, Hartford Hospital, Hartford, CT 06102, USA; ^2^University of Connecticut, Storrs, CT 06269, USA; ^3^University of Hartford, West Hartford, CT 06117, USA; ^4^University of Massachusetts, Amherst, MA 01003, USA

## Abstract

Statins are the most widely prescribed and effective medication for reducing low density lipoprotein cholesterol. Statins may also lower resting blood pressure (BP); however, results are inconsistent. We sought to determine if the maximum dose of atorvastatin reduces resting BP and the peak systolic BP (SBP) achieved on a graded exercise stress test (GEST) among a large sample of 419 healthy men (48%) and women (52%). Subjects (419, 44.1 ± 0.8 yr) were double-blinded and randomized to 80 mg·d^−1^ of atorvastatin (*n* = 202) or placebo (*n* = 217) for 6 mo. Among the total sample, there were no differences in resting BP (SBP, *P* = 0.30; diastolic BP [DBP], *P* = 0.69; mean arterial pressure (*P* = 0.76); or peak SBP on a GEST (*P* = 0.99)) over 6 mo, regardless of drug treatment group. However, among women on atorvastatin, resting SBP/DBP (3.7±1.5 mmHg, *P* = 0.01/3.2±0.9 mmHg, *P* = 0.02) and peak SBP on a GEST (6.5±1.5 mmHg, *P* = 0.04) were lower versus men. Atorvastatin lowered resting BP 3-4 mmHg and peak SBP on a GEST ~7 mmHg more among women than men over 6 mo of treatment. The inconsistent findings regarding the antihypertensive effects of statins may be partially explained by not accounting for sex effects.

## 1. Introduction

Statins are the most commonly prescribed and effective medication for reducing low density lipoprotein (LDL) cholesterol and, consequently, lowering cardiovascular disease (CVD) risk [1]. Interestingly, statins may produce other nonlipid, pleiotropic health benefits that may additionally lower CVD risk [2–8]. For example, they may decrease resting blood pressure (BP), which could have a substantial public health impact because hypertension affects one in three U.S. adults and one billion people worldwide [9, 10] and is a major risk factor for heart disease, stroke, congestive heart failure, and kidney disease [11, 12]. Indeed, a recent review has shown that statins lower systolic blood pressure (SBP) up to 8.0 mmHg in patients with dyslipidemia and normal BP; 6.0 mmHg in patients without dyslipidemia and with hypertension; and 13.7 mmHg in patients with dyslipidemia and hypertension [2]. However, other reports have reported no effect of statins on resting BP, and thus results are inconsistent regarding the influence of statin therapy on resting BP [13–15].

These inconsistencies could be attributable to a very small effect of statins on resting BP, such that the benefits of statins on BP may only be apparent during conditions in which BP is augmented, such as exercise. Although a hypertensive response to exercise is predictive of developing future hypertension and increases CVD risk, to the best of our knowledge the effect of statins on the peak SBP response to a graded exercise test (GEST) has never been examined [16].

Therefore, the purpose of the current analysis was to examine the influence of 80 mg of atorvastatin on resting BP and the peak SBP response to a GEST before and after 6 mo among healthy men and women ranging in age from 20 to 76 yr.

## 2. Methods

The present study is part of a larger clinical trial, “*The Effect of Statins on Skeletal Muscle Function and Performance*,” of which the methods have previously been published in detail (STOMP; NIH R01HL081893-01A2) [17, 18]. STOMP was the first randomly assigned, double-blinded study designed to examine the incidence of statin-induced muscle complaints, defined as myalgia, and the effect of statins on skeletal muscle strength and endurance and cardiorespiratory fitness among healthy adults across the lifespan [17].

STOMP investigated the effects of 80 mg of atorvastatin compared to placebo for 6 mo in healthy adults ranging from 20 to 76 yr. STOMP subjects were recruited by newspaper advertisement, local flyers, posters, and classroom announcements at each of the three testing sites. Prior to participating in the study, individuals were screened by telephone and signed a written informed consent approved by the Institutional Review Boards of the participating institutions. Healthy men and women were included if they were not previously on a statin, had normal thyroid function, and had no history of CVD, diabetes, or cancer within the previous 5 yr. Low density lipoprotein (LDL) cholesterol was not an inclusion/exclusion criterion in the main study as many patients with CVD or other cardiovascular risk factors presently receive statin therapy regardless of their baseline LDL values.

Height, weight, waist circumference, and fasting blood drawn to assess blood lipids-lipoproteins were obtained. Height and weight were measured and then used to calculate body mass index (BMI) (kg/m^2^). Resting BP and heart rate were measured following a seated rest period of 5 min by trained research personnel. Subjects then completed a GEST using the modified Balke protocol to determine peak oxygen consumption (VO_2peak_) [17, 19]. Participants were then double-blinded and randomized to receive 80 mg of atorvastatin (*n* = 202) or a placebo (*n* = 217) and instructed to take two capsules at night daily during the next 6 mo. Research personnel telephoned subjects every other week inquiring about changes in medication use, medication adherence to the study drug, and any symptoms of myalgia. Study drug compliance was calculated at 3 and 6 mo via pill capsule count. All study procedures (i.e., resting BP, fasted blood draw, and GEST) were repeated following 6 mo of drug treatment. The analysis of this substudy did not include subjects that reported myalgia in the main study as they would have posttested earlier than 6 mo.

### 2.1. Blood Lipids

Blood samples were obtained and lipids measured as previously described [17]. Briefly, samples were drawn before and after 6 mo of drug treatment from the antecubital vein for analysis of the lipid-lipoprotein profile (i.e., total cholesterol, LDL cholesterol, high density lipoprotein cholesterol (HDL), and triglycerides). LDL cholesterol was calculated using the Friedewald equation [20].

### 2.2. Resting Blood Pressure and Heart Rate

Resting BP was measured following a 5 min seated rest prior to the peak GEST. Subjects were seated with both feet flat on the floor, legs uncrossed, and backs supported. A trained research associate measured SBP and diastolic BP (DBP) by auscultation in the right arm supported at heart level according to standard procedures for BP assessment [21]. A heart rate monitor (Polar Vantage NV HR Monitor, Polar Electro Inc., Port Washington, NY, USA) measured resting heart rate. Mean arterial pressure (MAP) was calculated as MAP = ((2 × DBP) + SBP)/3).

### 2.3. Peak Cardiopulmonary Graded Exercise Test

VO_2peak_ was determined on a treadmill with a modified Balke protocol [17, 19]. The subjects sat for 5 min to establish gas equilibration at which time a pre-GEST resting BP was measured via auscultation in the right arm. The Parvomedics True One 2400 Metabolic Cart (ParvoMedics Corp, Sandy, UT) was used for breath-by-breath analysis of expired oxygen and carbon dioxide to determine VO_2peak_.

Subjects began the peak GEST by walking at a 2 mph on a 0% incline for 2 min. Following this warm-up, research personnel increased the treadmill speed to a pace that the subject could maintain to complete the test. The incline of the treadmill increased 1% every minute until the conclusion of the test. Heart rate, BP, and the Borg scale rating of perceived exertion were recorded [19]. The subject continued to exercise until they reached volitional fatigue, or if research personnel had to stop the test for any other reason such as the case if the subject felt they could not continue.

### 2.4. Peak Systolic Blood Pressure

The peak SBP achieved on the GEST was the highest SBP recorded 1 min prior to the end of exercise. If peak SBP could not be attained during exercise, peak SBP was obtained within 30 sec of exercise cessation. If SBP was measured at 1 min before and immediately after a GEST, the latter was used as peak SBP. Only 49 subjects had exercise peak SBP measurements obtained before and after the study due to excessive movement at peak exercise. These subjects serve as the subsample to examine the influence of statins on the peak SBP response to a GEST.

### 2.5. Statistical Analysis

Differences in baseline characteristics between placebo and atorvastatin groups were assessed with a 1-way analysis of variance (ANOVA) with significance set *P* < 0.05. Data are reported as mean ± standard error of the mean (SEM). Independent samples *t*-tests were used to compare baseline characteristics between men and women by atorvastatin and placebo groups. Bivariate correlations were run to test for significant relationships between changes in LDL cholesterol and changes in resting and peak SBP. Repeated measures analysis of covariance (ANCOVA) tested between-group differences in the change of resting BP and peak SBP on a GEST at 6 mo from baseline with sex, menopausal status, and BP medication use as fixed factors and age, BMI, and VO_2peak_ as covariates. Drug ∗ sex interactions were analyzed using ANCOVA with Bonferroni correction. All statistical analyses were performed using the Statistical Package for the Social Sciences (SPSS) 14.0 program for Windows (SPSS Inc, Chicago, IL).

## 3. Results

The total sample for this analysis (*n* = 419) included healthy men (*n* = 203) and women (*n* = 216) that were on average middle aged and overweight and had optimal resting BP, above optimal LDL cholesterol, desirable total cholesterol, and normal triglyceride levels (Tables 1 and 2) [1, 11, 22]. Cardiorespiratory fitness levels ranged from poor (women) to fair (men) when compared to age matched normative values (Table 1) [19]. Men had a greater BMI, triglyceride levels, VO_2peak_, and lower HDL cholesterol levels than women (all *P* ≤ 0.01) (Table 1). Resting SBP (*P* = 0.01), DBP (*P* = 0.04), MAP (*P* = 0.01), and peak SBP on a GEST (*P* < 0.001) were also higher among men compared to women (Tables 2 and 3). Women assigned to the atorvastatin group had a higher resting DBP (*P* = 0.04) than women assigned to the placebo group (Table 2). Among the total sample, 19 men (53%) and women (47%) were prescribed antihypertensive medications.

### 3.1. Resting Blood Pressure

Among the total sample, the change in resting SBP (*P* = 0.60), DBP (*P* = 0.96), and MAP (*P* = 0.74) was not different from baseline over 6 mo of treatment between the groups (Table 2). However, there were significant drug ∗ sex interaction effects for resting SBP (*P* = 0.02) and DBP (*P* = 0.02). Women on atorvastatin reduced resting SBP (*P* = 0.01) and DBP (*P* = 0.02) from baseline over 6 mo of drug treatment (Table 2), whereas the change in BP was not different among men on atorvastatin (*P* > 0.05). However, these sex-dependent atorvastatin BP effects were not different than placebo (SBP, *P* = 0.20; DBP, *P* = 0.60).

### 3.2. Peak Systolic Blood Pressure

Among the total sample, the peak SBP response on a GEST was not different over 6 mo of treatment, regardless of drug group (*P* = 0.99) (Table 3). However, there were significant drug ∗ sex interaction effects (*P* = 0.02). Among women on atorvastatin, peak SBP on a GEST decreased from baseline over 6 mo between the groups (*P* = 0.02) but was not different among women on placebo (*P* = 0.40) (Table 3).

### 3.3. Lipid-Lipoprotein Profile and Blood Pressure Correlations

Over 6 mo of drug treatment, LDL cholesterol (−58.9 ± 2.0 mg·dL^−1^), total cholesterol (−65.3 ± 2.2 mg·dL^−1^), and triglyceride levels (−28.3 ± 3.1 mg·dL^−1^) decreased among the total sample and atorvastatin group (*P* < 0.001), but not the placebo group (*P* > 0.05). Over 6 mo of drug treatment, there were no significant correlations among the changes of any component of the lipid-lipoprotein profile and resting or the change in SBP, DBP, MAP, and the peak SBP response to a GEST among the total sample or by drug group (all *P* > 0.05). Over 6 mo of drug treatment, there was a weak but significant correlation between the change in resting DBP and triglyceride levels (*r* = 0.154, *P* = 0.03) among the atorvastatin group.

## 4. Discussion

The purpose of the present study was to determine if the maximum dose of atorvastatin used to treat dyslipidemia reduces resting BP and the peak SBP achieved on a GEST. Among the total sample, there were no significant differences in resting BP, MAP, or peak SBP on a GEST over 6 mo, regardless of drug treatment group. However, atorvastatin lowered resting BP 3-4 mmHg and peak SBP on a GEST ~7 mmHg more among women than men over 6 mo of treatment. These results suggest that the pleiotropic effect of statins may be sex dependent and that previous inconsistencies in the literature regarding statins and BP may be attributable in part to these sex differences.

The mechanisms by which statins may reduce BP have been reported to involve the renin-angiotensin-aldosterone system (RAAS), the endothelial nitric oxide (eNOS) pathway, and the sympathetic nervous system (SNS) [2, 3, 7]. For example, hypercholesterolemia is associated with overexpression of the AT1 receptor, augmenting BP [23, 24]. Statins may reduce BP by downregulating AT1 receptor expression while increasing AT2 receptor activity. Similarly, statins increase the bioavailability of the vasodilator nitric oxide [23, 25], decrease the vasoconstrictor endothelin-1 (ET-1) expression, and reduce sympathetic nerve activity [26], all favorable vascular alterations which may serve to reduce BP after statin therapy.

The sex-specific effects of statins on BP may be attributable to the influence of estrogen on pathways mentioned above, such as the actions of statins within the RAAS [27]. Estrogen is known to downregulate the angiotensin-converting enzyme and the AT1 receptor, lowering BP [27]. Downregulation of the AT1 receptor may also activate the AT2 receptor, which promotes NO release [24] and improves endothelial function [28]. Therefore, it seems possible that synergistic actions of statins and estrogen to promote vasodilation via their counterregulatory actions on RAAS AT1 and AT2 receptors may explain the lower BP we observed both at rest and during peak exercise among women on atorvastatin but not men. Similarly, estrogen also influences control of BP through the autonomic nervous system by blunting the vascular response to SNS stimuli via adrenergic receptor buffering [29–31]. Premenopausal women have* less* *α*-adrenergic receptor control of resting BP and* greater* *β*2-adrenergic receptor-mediated vasodilation than men [32]. This could also explain sex-dependent effects of both resting and the peak exercise BP-lowering effects of atorvastatin, although we caution that the small numbers of patients with a peak SBP on a GEST (*n* = 49) in the current analysis warrant cautious interpretation of these data. Furthermore, menopausal status at the time of enrollment was not a significant factor in the present study.

There are several limitations to the substudy. First, the present study consists of a post hoc analysis of the larger STOMP trial [17] and thus was not originally powered to examine resting BP or the peak SBP response to a GEST as major outcomes. However, with 200 patients on atorvastatin and a power of 0.80, we were powered to detect a difference of 0.3 mmHg in resting or peak SBP on a GEST at an alpha of 0.05, suggesting that negative findings in men and the overall sample were not due to type II statistical error. We also reduced variability in the BP response by examining the effect of a single, maximum daily dose of atorvastatin (80 mg) for 6 mo versus placebo with very stringent BP assessment performed in a laboratory setting both at rest and at peak exercise [21]. Prior studies used statins of various doses and types and less rigorous assessments of BP, which may also contribute to heterogeneity in the published literature regarding the influence of statins on BP. Second, we lacked key measurements of biomarkers that could possibly explain our findings (i.e., angiotensin II, aldosterone, NO, ET-1, and estrogen); therefore, the proposed mechanisms are purely* speculative*. Third, the rubric used to determine the peak SBP on a GEST yielded 49 participants of the total sample, and thus these findings are preliminary and warrant further investigation. Lastly, although we did not detect cholesterol-dependent effects of statins on BP, the lack of correlation is not strong enough to rule out this potential mechanism. This is likely due to the fact that LDL cholesterol was not an inclusion/exclusion criterion in the main study. Given that the subjects were healthy and would likely not be receiving statin therapy, these results may not be representative of this population. However, many patients with CVD or other CVD risk factors presently receive statin therapy regardless of BP and LDL levels.

Statins are the most widely prescribed medication in the world, while hypertension affects one billion people worldwide [33]. Given that hyperlipidemia and hypertension commonly coexist [34], the effectiveness of statins as a single pharmacological intervention to reduce both cholesterol and BP is clinically intriguing. The use of statins as monotherapy to treat coexisting conditions may benefit some populations by reducing patient costs and potential side effects as well as improving drug adherence [35, 36]. Our findings are supportive of the nonlipid, pleiotropic health benefit of statins to resting BP and the peak SBP response on a GEST, especially among women. However, further investigation is needed with a randomized controlled trial intentionally designed to determine the influence of statins on BP among men and women with hypertension to confirm the sex-dependent effects we observed.

## Figures and Tables

**Table 1:** Baseline subjects characteristics (mean ± SEM) among the total sample and by atorvastatin and placebo groups by sex.

Variable	Total sample (*n* = 419)	Atorvastatin (*n* = 202)		Placebo (*n* = 217)	
		Men (*n* = 99)	Women (*n* = 103)	Men (*n* = 104)	Women (*n* = 113)
Age (yr)	44.1 ± 0.8	43.5 ± 1.6	43.3 ± 1.6	43.6 ± 1.5	45.6 ± 1.6
BMI (kg*·*m^−2^)	26.4 ± 0.2	27.4 ± 0.4	25.2 ± 0.5^**^	27.4 ± 0.4	25.6 ± 0.5^*^
LDL (mg*·*dL^−1^)	117.4 ± 1.6	121.3 ± 3.1	116.6 ± 3.9	118.6 ± 2.8	113.5 ± 3.2
Total cholesterol (mg*·*dL^−1^)	196.6 ± 1.9	196.5 ± 3.3	200.7 ± 4.4	191.6 ± 3.2	197.7 ± 3.8
HDL (mg*·*dL^−1^)	58.1 ± 0.8	49.9 ± 1.4	65.0 ± 1.6^†^	50.6 ± 1.1	66.0 ± 1.7^†^
Triglycerides (mg*·*dL^−1^)	106.6 ± 2.7	126.0 ± 6.3	95.6 ± 4.1^†^	112.3 ± 6.2	94.6 ± 4.3^†^
VO_2peak _(mL*·*kg^−1^ *·*min^−1^)	33.9 ± 0.5	38.6 ± 0.9	30.9 ± 0.9^†^	37.8 ± 0.9	29.0 ± 0.8^†^
Blood pressure medication use	*n* = 19	*n* = 3	*n* = 5	*n* = 7	*n* = 4

BMI, body mass index; HDL, high density lipoprotein; LDL, low density lipoprotein; HR, heart rate; VO_2peak_, peak oxygen consumption.

^*^ *P* < 0.05, _ _^**^ *P* < 0.01, and ^†^ *P* < 0.001; men versus women.

**Table 2:** Average adjusted resting blood pressure change (mean ± SEM) after 6 mo of 80 mg atorvastatin or placebo among total sample and by drug and sex.

Resting BP (mmHg)	Total sample		Atorvastatin		Placebo	
	Pre	Δ	Pre	Δ	Pre	Δ
Total	*n* = 419		*n* = 202		*n* = 217	
SBP	118.9 ± 0.6	0.5 ± 0.5	115.3 ± 0.9	0.8 ± 0.7	114.7 ± 0.8	0.3 ± 0.6
DBP	75.3 ± 0.5	0.3 ± 0.5	75.5 ± 0.6	0.4 ± 0.7	75.2 ± 0.6	0.5 ± 0.6
MAP	87.7 ± 0.7	0.2 ± 0.8	88.5 ± 0.8	0.0 ± 0.8	86.8 ± 1.1	0.5 ± 1.3
Men	*n* = 203		*n* = 99		*n* = 104	
SBP	117.2 ± 0.8	1.2 ± 0.7	117.7 ± 1.2	2.7 ± 1.0	116.6 ± 1.1	−0.2 ± 1.0
DBP	76.5 ± 0.6	1.0 ± 0.7	75.0 ± 0.9	2.0 ± 1.0	77.0 ± 0.9	0.2 ± 0.9
MAP	89.3 ± 1.0	1.2 ± 1.0	90.1 ± 0.9	0.9 ± 1.2	87.7 ± 1.6	1.7 ± 1.8
Women	*n* = 216		*n* = 102		*n* = 113	
SBP	113.0 ± 0.8^*^	−0.1 ± 0.7	113.0 ± 1.2^*^	−1.0 ± 1.1^*^	113.0 ± 1.2^*^	0.8 ± 0.8
DBP	74.3 ± 0.6^*^	−0.4 ± 0.7	76.0 ± 0.9^*^	−1.2 ± 0.9^*^	73.4 ± 0.9^∗‡^	0.8 ± 0.9
MAP	86.1 ± 0.9^*^	−0.7 ± 1.2	86.9 ± 0.9^*^	−0.8 ± 1.2	85.9 ± 1.5^*^	−0.7 ± 1.8

Adjusted for age, baseline BMI, baseline VO_2peak_, and sex.

Pre, baseline; Δ, after 6 months; SBP, systolic blood pressure; DBP, diastolic blood pressure; MAP, mean arterial pressure.

^*^ *P* < 0.05; men versus women.

^‡^ *P* < 0.05, atorvastatin versus placebo.

**Table 3:** Average adjusted peak systolic blood pressure (mean ± SEM) to a GEST after 6 mo of 80 mg atorvastatin or placebo among total sample and by drug and sex.

True max peak SBP (mmHg)	Total sample		Atorvastatin		Placebo	
	Pre	Δ	Pre	Δ	Pre	Δ
Total	*n* = 196		*n* = 98		*n* = 98	
	169.6 ± 1.1	0.2 ± 1.1	169.0 ± 1.6	−0.7 ± 1.6	169.9 ± 1.7	0.6 ± 1.4

Men	*n* = 105		*n* = 49		*n* = 56	
	174.9 ± 1.9	0.7 ± 1.5	175.4 ± 2.6	2.5 ± 2.4	177.7 ± 2.3	−0.9 ± 1.8

Women	*n* = 91		*n* = 49		*n* = 42	
	164.5 ± 2.0^*^	−0.9 ± 1.6	162.6 ± 2.6^*^	−3.9 ± 2.2^*^	162.1 ± 2.8^†^	2.6 ± 2.2^‡^

Adjusted for age, baseline BMI, baseline VO_2peak_, and sex.

Pre, baseline; Δ, after 6 months; SBP, systolic blood pressure; GEST, graded exercise stress test.

^*^ *P* < 0.05, ^†^ *P* < 0.001; men versus women.

^‡^ *P* < 0.05, atorvastatin versus placebo.
